# Development and performance assessment of a novel scroll compressor-based oxygen generator integrated ventilator

**DOI:** 10.1038/s41598-025-94363-w

**Published:** 2025-03-21

**Authors:** Xiaokang Yu, Jing Yan, Lijun Ruan, Mingzhi Luo, Bo Che, Linhong Deng, Yuxi Luo

**Affiliations:** 1 School of Biomedical Engineering, Shenzhen Campus of Sun Yat-Sen University, Shenzhen, 518000 Guangdong China; 2 Institute of Biomedical Engineering and Health Sciences, Changzhou University, Changzhou, 213000 Jiangsu China; 3 Key Laboratory of Sensing Technology and Biomedical Instruments of Guangdong Province, Sun Yat-Sen University, Shenzhen, 518000 Guangdong China

**Keywords:** Mechanical ventilation, Pressure swing adsorption, Scroll compressor, Oxygen generator integrated ventilator, Biomedical engineering, Asthma, Chronic obstructive pulmonary disease, Respiratory distress syndrome

## Abstract

Current ventilators rely on wall outlets or cylinders for oxygen supply, which limits their continuous use in the field or emergencies. In this study, we proposed a ventilator prototype that can achieve stand-alone oxygenated respiratory support, by designing and integrating a high-performance oxygen generator, and optimizing the control strategies of the whole system. Based on the designed oil-free scroll compressor and pressure swing adsorption (PSA) system, we first realized a mobile high-flow oxygen generator, which achieved an output flow greater than 17 L/min with an oxygen concentration of 93% ± 3%. The ventilator was also designed to synchronize with the respiratory state, to optimize the trigger performance for the pressure support of early inspiration, and reduce the gas supply in the late inspiratory phase to avoid pressure overshoot in the early expiratory phase. The respiratory synchronization of the integrated ventilator was estimated by the recorded chest movement of the subjects. Satisfactory respiratory synchronization was realized with an inspiratory trigger delay (ITD) time of less than 200 ms and sound respiratory waveform tracking. By regulating the PSA strategy, the oxygen generation and utilization efficiencies could be further improved. Ultimately, under the setting of inspiratory positive airway pressure (IPAP) at 10 cmH_2_O, and expiratory positive airway pressure (EPAP) at 4 cmH_2_O, we achieved non-invasive ventilation with a maximum oxygen concentration of 58% ± 1.75%. In conclusion, the proposed oxygen generator integrated ventilator could provide reliable oxygenated respiratory support in emergencies, such as on-site first aid, patient transport, and military field environments.

## Introduction

Mechanical ventilators play a pivotal role in saving the lives of patients with respiratory failure, by providing high concentration oxygen to maintain open alveoli, reduce respiratory muscle burden, and thus improve patient oxygenation^1–4^. Existing non-invasive ventilators are supplied with oxygen from either oxygen cylinders or centralized oxygen stations in the hospital^5^. In emergencies, such as first aid or patient transport, a convenient and sustainable oxygen supply is critically important^6^. However, medical experts of public emergencies have highlighted the significant shortage of mechanical ventilation and oxygen support in emergency medical treatment^7–9^, particularly the hyperbaric oxygen sources used to support the operation of ventilators or anesthesia machines^10,11^.

In mobile settings, ventilators are supplied with oxygen from oxygen cylinders, but the processes of filling, maintaining, and transporting cylinders constrain the application of oxygenated respiratory support to a certain extent^12^. A safer and more convenient option of providing continuous oxygen sources for viable ventilators is to employ mobile pressure swing adsorption (PSA) oxygen generators^13^. Nevertheless, the current mobile PSA oxygen generators are limited in oxygen generation capacity and output pressure, making it difficult to achieve high oxygen output in emergency situations. Therefore, some researchers are dedicated to developing a hyperbaric oxygen source for non-invasive ventilators, and our collaborators have proposed a group standard for mobile high-pressure oxygen supply systems in China. The key factors influencing the oxygen output for PSA oxygen generators are the compression capacity of the compressor and the PSA parameters of the molecular sieve^14^. In PSA technologies, the purge flow rate and adsorption pressure have been optimized under various conditions, which improves the oxygen purity, yield coefficient, and productivity^15^. The efficiency of adsorption and desorption processes can be enhanced by using nano-zeolite with a high surface-area-to-volume ratio in place of molecular zeolites^16^. Regarding the compressor, Nair et al. showed that increasing the length of the piston cylinder and reducing the inner diameter can increase the intake volume and reduce airflow resistance, thus enhancing efficiency and oxygen concentration^17^. Rubio J et al. realized a flow rate of 15 L/min and an oxygen concentration of 93% with an oxygen generator that connected two oxygen generators in parallel^18^. They noted that such a high-flow oxygen generator can supply oxygen to some ventilators, thereby assisting patients in maintaining life through mechanical ventilation. Due to their excellent characteristics, scroll compressors are widely used for liquid compression in various fields^19^. Oil-free scroll compressors are also used in large-scale oxygen generation equipment^20^. We have developed a miniaturized oil-free scroll compressor for gases through improvements in machining precision and the alignment of orbiting and fixed scrolls, with which a high-performance oxygen generator has been designed in this study.

We also developed a control strategy for subject-ventilator synchronization for the proposed oxygen generator integrated ventilator because it is always a crucial issue in the clinical application of mechanical ventilation. The lack of synchronization may lead to prolonged periods of assisted ventilation, increasing the work of breathing, and other complications^21–23^. High levels of trigger sensitivity and the performance of pressure rise during the inspiratory phase are essential to ensure efficient ventilation in clinical scenes^24^. Flow-based triggering enables the ventilator to accurately identify the patient’s respiratory state and provide appropriate pressure support in a timely manner.

The objective of this study was to develop a ventilator that can achieve stand-alone oxygenated respiratory support without an external oxygen source, providing ventilation with oxygen concentration close to 60% for adults. To this end, we first achieved an outperforming oxygen generation module by using a designed oil-free scroll compressor. In comparison to existing oxygen generators of similar size and weight, this module improved the oxygen generation capacity by nearly 70%, from 10 L/min to 17 L/min. Further, we optimized the oxygen supply strategy and PSA parameters to increase the maximum oxygen concentration delivered by the proposed ventilator. It achieved an oxygen concentration of 58% under bi-level positive airway pressure (BiPAP) conditions, representing a 20% increase relative to the direct output of a 10 L/min oxygen generator in conjunction with a ventilator. In addition, the subject-ventilator synchronization was also designed and estimated based on respiratory phase sensing. This innovative machine holds significant promise for the treatment of critically ill patients in emergency medical rescue scenarios.

## Methods

### System architecture

**Fig. 1:**
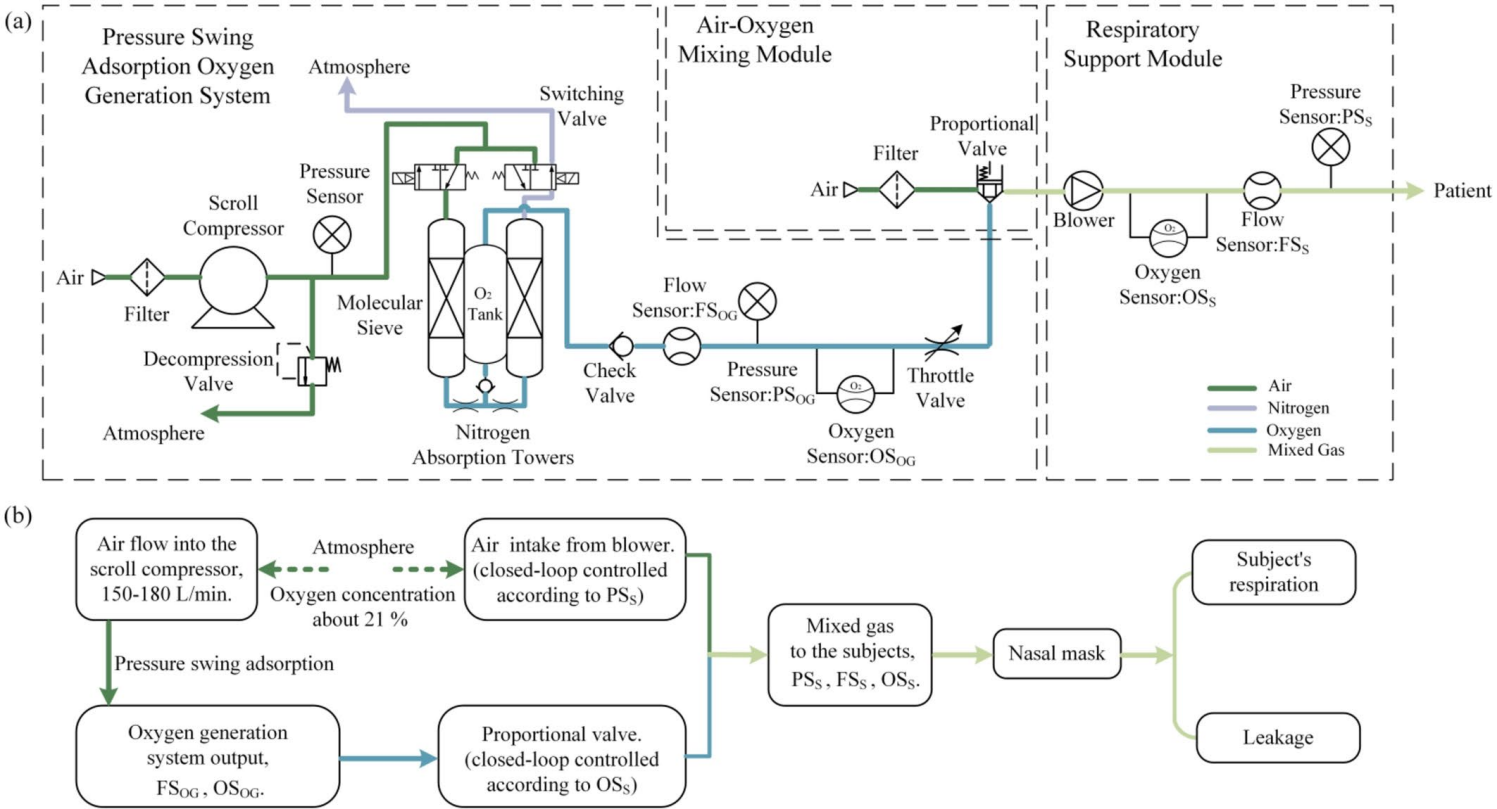
(**a**) Schematic diagram of the oxygen generator integrated ventilator; (**b**) Flow composition diagram.

The structure of the proposed oxygen generator integrated ventilator is shown in Fig. 1(a). It mainly includes the scroll compressor-based PSA oxygen generation system, air-oxygen mixing module, and respiratory support module.

The flow composition of the system is shown in Fig. 1(b). The filtered air is initially introduced into the oil-free scroll compressor. Then, compressed air enters the molecular sieve tower for nitrogen adsorption. Two towers work alternately, when one tower generates high oxygen concentration air into the oxygen storage tank, the other tower exhausts the adsorbed nitrogen into the atmosphere to reactivate the molecular sieve. A servo proportional valve is used to control the flow rate of high oxygen concentration air into the next module. In the respiratory support module, a brushless DC blower draws air from the atmosphere to the patient, creating a certain support pressure through a nasal mask. The oxygen concentration of the respiratory support air is regulated by the mixing ratio of the air from the blower and the high oxygen concentration air from the oxygen storage tank. The flow sensor in the respiratory support module is applied to obtain respiratory waveform and to determine the inspiratory or expiratory phase. In the end, since we are using non-invasive ventilation, part of the air passing through the nasal mask is inhaled by the subject and the other part leaks into the atmosphere.

**Fig. 2:**
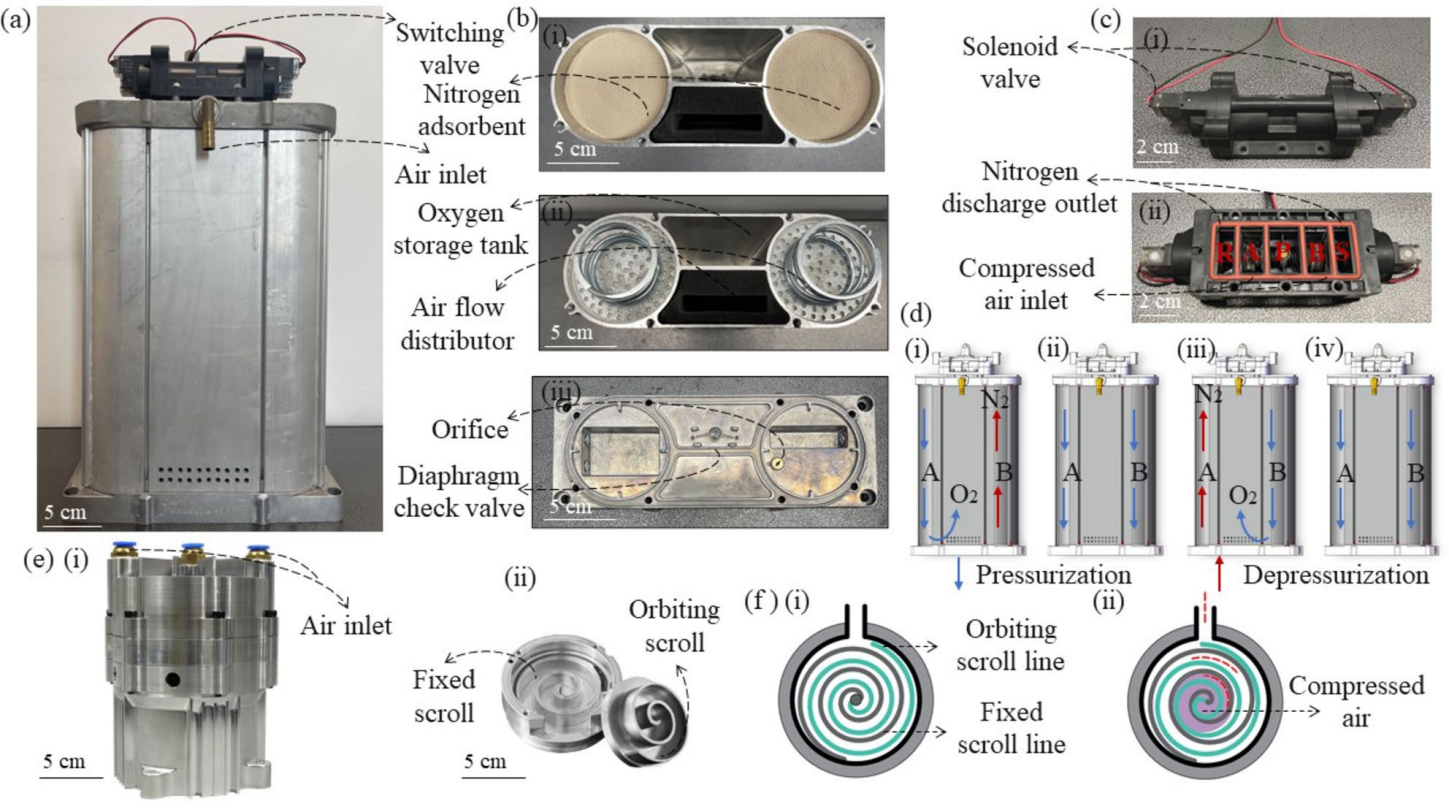
Oil-free scroll compressor-based pressure swing adsorption oxygen generation system: (**a**) Molecular sieve towers, (**b**) Filling process of molecular sieve, (**c**) Structure of the switching valve, (**d**) Steps in the cycle of PSA oxygen generation, (**e**) Structure of the oil-free scroll compressor, (**f**) Gas compression process of the scroll compressor.

The core components of our PSA oxygen generation system are shown in Fig. 2. This module mainly comprises molecular sieve towers in Fig. 2(a), working condition switch valves in Fig. 2(c) including an integrated valve composed of two electrically controlled three-way valves and pressure check valves (a designed integral diaphragm), a designed oil-free scroll compressor in Fig. 2(e). The main innovation of the oxygen generation system is the use of a scroll compressor. The absence of mechanical friction confers it a number of advantages over traditional piston compressors, including high compression efficiency, high gas output, low noise, and low vibration. As shown in Fig. 2(f), in a scroll compressor, air is drawn into the spiral cavity, which narrows as the orbiting scroll continues to rotate, thereby compressing the air. The orbiting scroll and the fixed scroll should move relatively without collision, and the gap between them is sufficiently small to ensure effective dynamic sealing and the formation of a compression cavity. These significantly increase the challenges associated with the design, manufacturing, and miniaturization of scroll compressors, particularly for gases. By optimizing the processing, structure, and materials, we have successfully developed an oil-free scroll compressor that can achieve discharge flow rates of up to 180 L/min with a weight of only 6 kg.

The molecular sieve towers are filled with lithium-type zeolite molecular sieve (Fig. 2(b)(i)), which exhibits a high nitrogen adsorption capacity when pressurized. PSA with twin towers requires four steps to realize continuous oxygen generation, as shown in Fig. 2(d). In steps (i) and (iii), one tower is fed with compressed air and pressurized to adsorb nitrogen, while the oxygen in the compressed air passes through the molecular sieve. The majority of the oxygen is directed to the oxygen storage tank, while a minor quantity is conveyed into another tower via a throttling passage situated at the base. This tower is connected to the atmosphere, allowing the adsorbed nitrogen to be released, while the above-mentioned small oxygen flow helps reactivate the molecular sieve. The check valves serve to ensure that only pressurized oxygen flow is permitted to enter the oxygen storage tank. We define the duration of each step (i) or (iii) as pressurization time. Steps (ii) and (iv) are short-period working conditions, which are defined as pressure equalization time. During these periods, two towers are both connected to the compressed air to achieve identical pressure distribution. The pressure equalization process balances the pressure difference between the adsorption and desorption processes, improving the reactive efficiency of the adsorbent. Such a pre-pressurization process also allows the adsorption tower to quickly reach working pressure. The switching of these four working steps is controlled by 2 two-position three-way solenoid valves. Additionally, the flow area of the valves affects both the pressurization and pressure equalization processes.

**Fig. 3:**
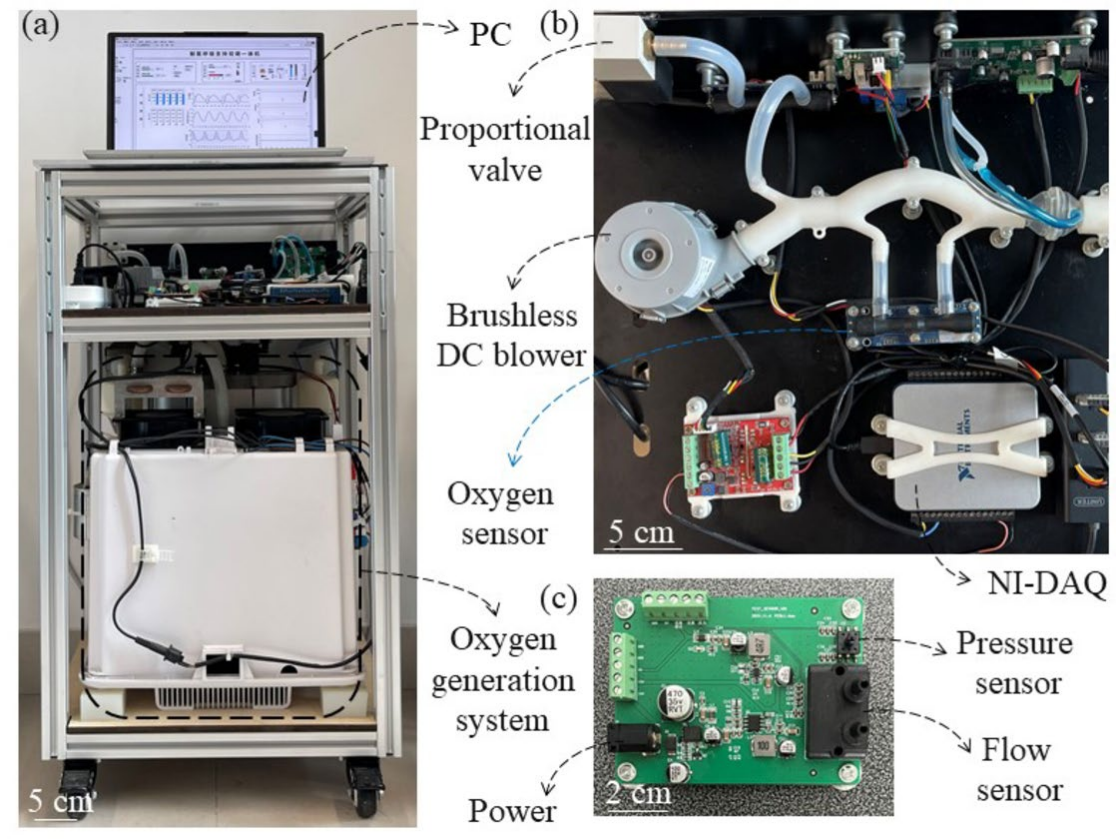
A prototype of the oxygen generator integrated ventilator. (**a**) The overall structure of the integrated ventilator. (**b**) Respiratory support system. (**c**) Printed circuit board with integrated flow and pressure sensors.

In Fig. 3(a), we designed a prototype of an integrated ventilator. It is difficult to achieve sufficient respiratory pressure support solely using the generated oxygen flow, additional air should be supplemented with a blower. Meanwhile, managing various oxygen concentrations also requires a controlled mix of air and the generated oxygen. Therefore, the air provided by the blower and the generated oxygen flow collectively constitute respiratory pressure support with a specific oxygen concentration. In this study, we designed BiPAP respiratory support, which means setting inspiratory positive airway pressure (IPAP) and expiratory positive airway pressure (EPAP) during inspiration and expiration, respectively. Since the dimensions of the current ultrasonic sensor are incompatible with the size of the respiratory support air passage, an airflow bypass for oxygen concentration detection has been designed, as shown in Fig. 3(b). In respiratory support module, a throttling differential pressure flow sensor as shown in Fig. 3(c) is applied to obtain respiratory curves. This real-time respiratory flow is the basis for identifying the inspiration and expiration. Pressure closed-loop control is achieved through a pressure sensor and a brushless DC blower, with a target pressure of IPAP or EPAP. An ultrasonic oxygen concentration sensor is employed to monitor the oxygen concentration of the respiratory support air, according to which we adjust the opening of the electromagnetic proportional valve to limit the generated oxygen flow rate entering the respiratory module.

### Control strategy

**Fig. 4:**
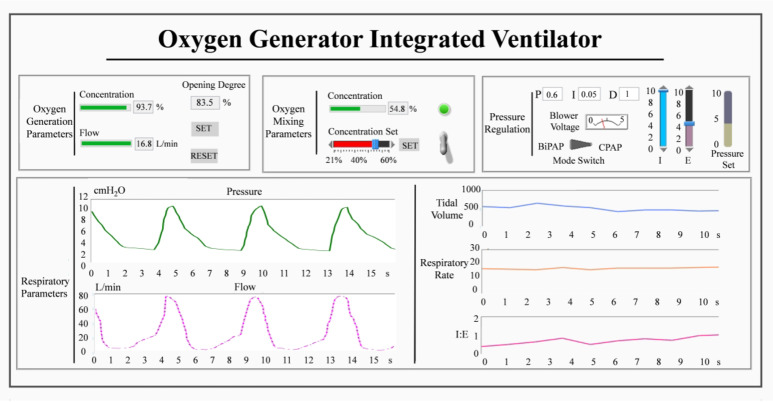
User interface of the proposed integrated ventilator.

The program was developed using LabVIEW 2017. A data acquisition (DAQ) device (NI USB-6001) and a PC constitute the hardware of the controller. The controller mainly includes four sub-tasks: (1) detection of respiratory phase; (2) closed-loop control of respiratory support pressure; (3) oxygen concentration control; and (4) operating condition adjustment of the oxygen generation. In terms of the user interface, we implemented the real-time calculation and display of respiratory parameters, as shown in Fig. 4.

**Fig. 5:**
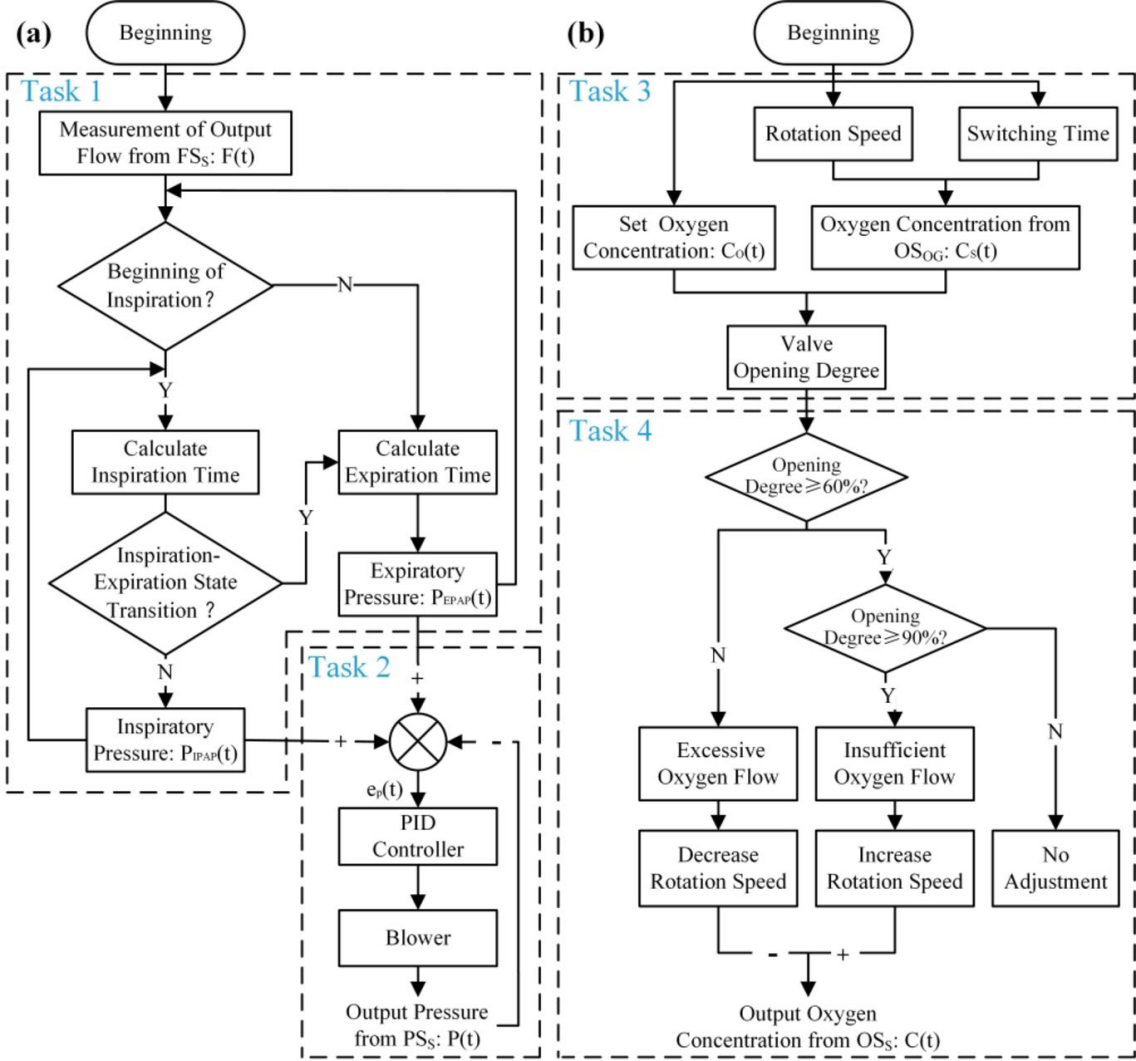
Control flowchart of the prototype. (**a**) Pressure control strategy. Task 1: detection of respiratory phase. Task 2: closed-loop control of respiratory support pressure. (**b**) Oxygen concentration control strategy. Task 3: oxygen concentration control. Task 4: operating condition adjustment of the oxygen generation.

The BiPAP helps open the airway, facilitates gas flow, reduces respiratory muscle stress, and improves ventilatory effect. The key to achieving such a ventilation pattern is identifying the subject’s inspiratory and expiratory phases, as shown in Fig. 5(a). By analyzing the respiratory flow in real-time, our ventilator is triggered to enter the inspiratory phase when a sudden increase in inspiratory airflow is detected. The trigger condition requires that the inspiratory flow rate exceeds 10 L/min and that the second derivative of the flow rate is greater than zero^25,26^. Due to the inertia delay of the blower, pressure overshoot will occur if the working condition switches at the actual beginning of expiration, which deteriorates subject-ventilator synchronization^27^. Therefore, the controller is programmed to enter the expiratory condition in advance. Through experimentation, an easily identifiable point, the maximum flow point of inspiration has been selected for this purpose. This means that when the inspiratory flow begins to decline, the controller enters the expiratory condition, as shown in Task 1 of Fig. 5(a).

The target control pressures for inspiration and expiration are set to IPAP and EPAP, respectively. The Proportional-Integral-Derivative (PID) controller regulates the brushless blower based on the difference between the set pressure and the actual pressure monitored by the pressure sensor to achieve closed-loop control, as shown in Task 2 of Fig. 5(a). In this study, we achieved oxygenated non-invasive ventilation by using a PSA oxygen generation system based on a scroll compressor. Under a certain PSA system, the oxygen output is determined by the rotation speed of the scroll compressor and the switching time of the above-mentioned working steps. As shown in Task 3 of Fig. 5(b), when the set oxygen concentration does not exceed the maximum oxygen generation capacity of the system, the oxygen generator provides an appropriate margin of oxygen generation and limits the generated oxygen flow through the proportional valve opening to achieve oxygen concentration control.

We also designed the adjustment logic of the oxygen generator to ensure it operates under suitable working conditions. As shown in Task 4 of Fig. 5(b), when the valve is open to a degree below 60%, indicating an excess supply of oxygen flow, we reduce the speed of the scroll compressor. Conversely, when the opening degree is greater than 90%, it indicates that the generated oxygen supply is insufficient. In this case, it is necessary to increase the rotation speed of the scroll compressor appropriately. This adjustment is performed once per minute in 25 rpm steps, approximately 2 L/min of the compressor flow output with each step.

### Functional evaluation

This section mainly includes 3 parts: (1) obtaining the relationship between the input and output parameters of the oxygen generator, (2) testing the oxygen concentration control and the maximum concentration of the support air under certain respiratory support parameters, (3) optimizing and estimating the subject-ventilator respiratory synchronization of the proposed integrated ventilator.

#### Performance of oxygen generator

The molecular sieve towers alternately adsorb nitrogen under pressurized conditions and desorb during the depressurization process, realizing continuous high-oxygen air generation. The main output indicators are the flow rate and oxygen concentration. Under optimized hardware conditions, the oxygen generation performance is contingent upon the output of the scroll compressor and working condition switching timing controlled by switching valves. By a throttle valve, the output flow rate was adjusted to 10–25 L/min, and the rotation speed was set to be 1500 rpm, 1800 rpm, and 2000 rpm, the output oxygen concentration of the system was estimated, so as to determine the optimal switching time of pressurization and pressure equalization. Under the optimal time sequence, the oxygen concentration of the system was further measured at output flow rates of 0–70 L/min. The relationship between output flow rates and oxygen concentration was applied to match the working conditions of the proposed integrated ventilator.

#### Oxygen concentration assessment

In this section, we focus on two functional evaluations: (1) ascertaining the maximum oxygen concentration that can be delivered by the integrated ventilator during noninvasive ventilation; (2) evaluating the stability of output oxygen concentration and testing the working condition matching function of the oxygen generation system.

For evaluation 1), to demonstrate the advantages of the scroll compressor-based oxygen generation system and the generation strategy designed for the integrated ventilator, we conducted ventilation experiments on subjects at generated output flow rates of 10 L/min, 17 L/min, and 26 L/min. These flow rates correspond to the maximum flow rate of the mobile oxygen generation system currently available on the market, the flow rate of the oxygen generation system at 93% oxygen concentration, and the flow rate when it outputs the maximum oxygen content, respectively.

The oxygen concentration and subject-ventilator synchronization performance of the integrated ventilator are related to respiratory parameters. Accordingly, we enrolled four healthy volunteers, two males, and two females. And informed consent was obtained from all participants. They were all 22 years old, weigh 71 kg, 76 kg, 53 kg, and 63 kg, height 174 cm, 171 cm, 162 cm and 166 cm, respectively. In evaluation 1), the oxygen generation system output the above three types of oxygen flows. In the testing of oxygen concentration control (evaluation 2)), the controlled oxygen concentration was set to 50%. The target IPAP and EPAP were set at 10 cmH_2_O and 4 cmH_2_O, respectively, for both sitting and supine postures. It means we conducted ventilation experiments in eight situations, each with a duration of five minutes, about 60–70 respiratory cycles.

#### Subject-ventilator synchronization

**Fig. 6:**
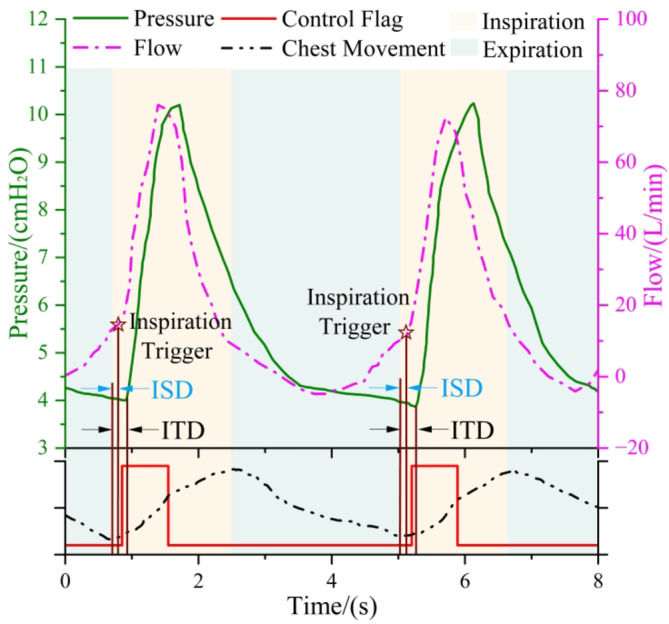
A demonstration of respiratory synchronization estimation. The star marks the trigger moment for the blower to begin to provide IPAP, and two-color blocks are used to distinguish the subjects’ inspiratory and expiratory phases.

As previously stated, the controller identifies the commencement of the inspiratory or expiratory phases based on the flow signal and sets the target pressure to IPAP or EPAP accordingly. The actual respiratory movements were simultaneously recorded using respiratory inductive plethysmography (RIP). The troughs and peaks of the RIP signal correspond to the beginnings of actual inspiration or expiration, respectively. As shown in Fig. 6, we display the RIP curve, control flag, flow, and pressure curves obtained by the sensors of the ventilator, to facilitate understanding of the time relationship between the control flag and the actual respiratory situation, as well as the synchronization of flow, pressure, and respiration. The time interval between the control flag transition and the actual onset of inspiration is designated as the inspiratory sensing delay (ISD). The inspiratory effort of the subject decreases the pressure of the ventilator, while the blower needs a response time. As a result, the pressure rise time will be later than the control flag setting time. Traditionally, scholars define the time difference between the actual beginning of inspiratory and the significant increase in pressure as inspiratory trigger delay (ITD) to assess inspiratory synchronization.

We estimate two aspects of respiratory synchronization: (1) ITD under various ventilation. Data are presented as mean value ± standard deviation (SD) if not stated otherwise. (2) The synchronization between the actual respiratory movement and the pressure support and flow rate curves provided by the ventilator.

## Result

### Oxygen generation system performance

In this section, the switching time of the oxygen generation system was first optimized. Subsequently, the relationship between flow and oxygen concentration at the optimal switching time was investigated.

**Fig. 7:**
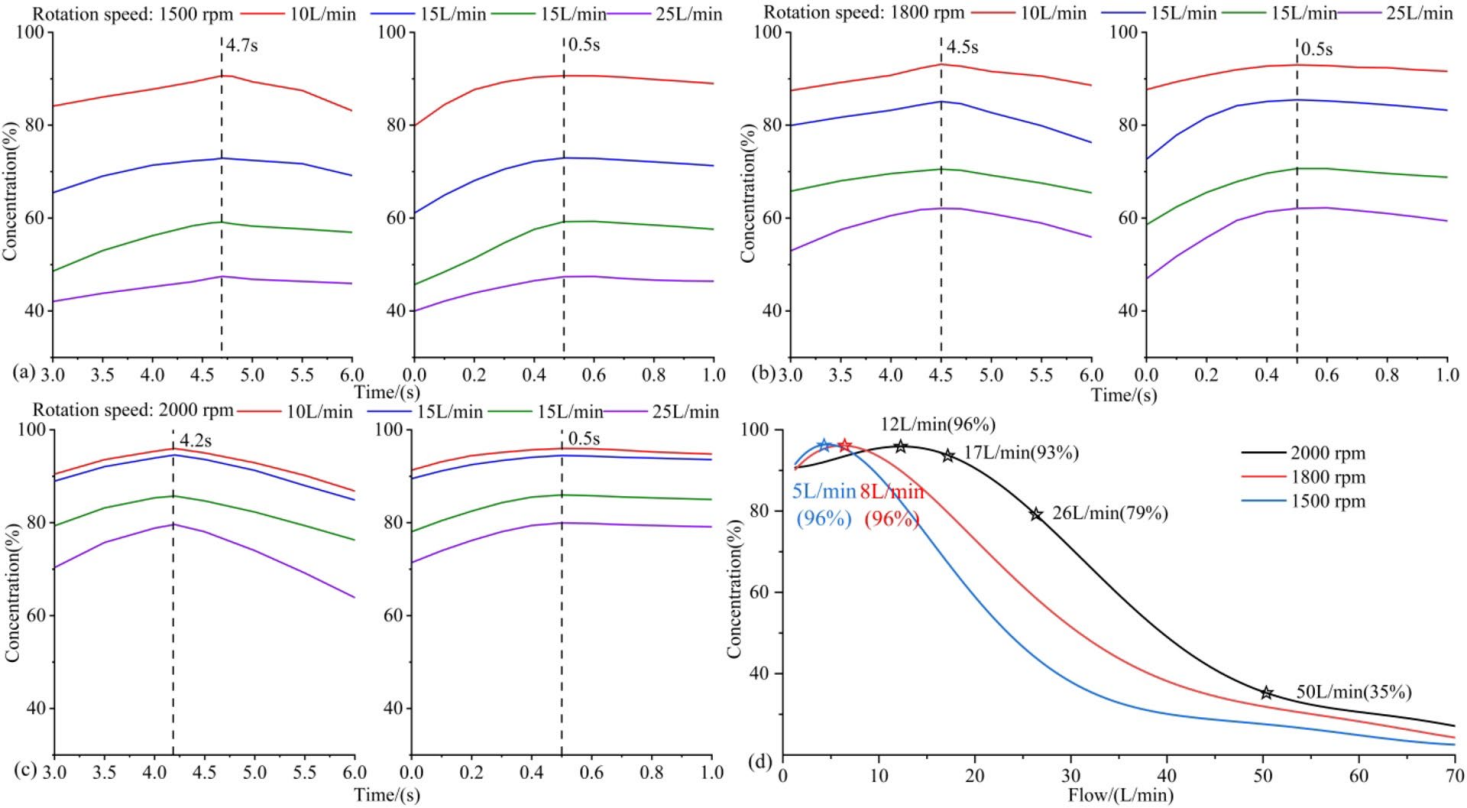
The output of the oxygen generation system. (a) Effect of switching time and flow rate on oxygen concentration at 1500 rpm of the compressor. The left and right figures respectively show the effects of pressurization and pressure equalization times. (b) Effect of switching time and flow rate on oxygen concentration at 1800 rpm of the compressor. (c) Effect of switching time and flow rate on oxygen concentration at 2000 rpm of the compressor. (d) The relationship between output flow rate and oxygen concentration at each of these 3 optimal switching times.

Different scroll compressor rotation speeds indicate different pressure change processes for pressurization adsorption, so we searched for the optimal switching time at compressor speeds of 1500 rpm, 1800 rpm, and 2000 rpm, respectively. At the same time, we also referred to the optimization methods of pressure switching time from previous studies^28–30^. The experimental results demonstrated that the optimal pressurization time and the optimal pressure equalization time are not obviously affected by the flow rates. Consequently, we employed a method of fixing one parameter to search for the optimal value of the other. The optimal switching times at the above rotation speeds were found to be 4.7s/0.5s, 4.5s/0.5s, 4.2s/0.5s, and the results are presented in Fig. 7(a)-(c). We demonstrated the effects of pressurization time and pressure equalization time on oxygen generation performance in the flow range of 10–25 L/min. In the optimization of pressurization time, the pressure equalization times were fixed to be 0.5 s. When we estimated the influence of pressure equalization time, the pressurization times were respectively set to be 4.7 s, 4.5 s and 4.2 s, corresponding to their optimized values. When ascertaining the maximum oxygen concentration for respiratory support that can be achieved by the proposed integrated ventilator, we should prioritize that the oxygen generation system provides as much mass value of oxygen as possible, rather than focusing solely on the maximum oxygen concentration. Therefore, the relationship between the output flow rate and oxygen concentration at each of the three optimal switching times was obtained, and the results are shown in Fig. 7(d).

Figure 7(a)-(c) present that the effects of pressurization time and pressure equalization time on oxygen concentration trends are similar across a range of compressor rotation speeds and flow rates. As the pressurization and pressure equalization time increases, the oxygen concentration first increases and then decreases, with a maximum value respectively occurring during the process. The time corresponding to this maximum value represents the optimal switching time under the given working conditions. Moreover, as the compressor rotation speed increases, the discrepancy in oxygen concentration between different output flow rates diminishes. When the rotation speed is set to 2000 rpm, in accordance with the prevailing concentration standard of 93 ± 3% for the mobile oxygen generation systems, the flow rate of the scroll compressor-based oxygen generation system is 17 L/min. On account of the concentration of oxygen at different flow rates, the relationship between the oxygen content of the output of the system and the flow rate can be obtained, which determines that the output flow rate corresponding to the highest oxygen content is 26 L/min, at which time the concentration of the oxygen output from the oxygen generation system is 79%.

### Oxygen concentration when ventilation

The oxygen concentration of the proposed integrated ventilator was tested with IPAP and EPAP set to 10 cmH_2_O and 4 cmH_2_O, respectively. The output flow rate of the oxygen generator was set to 10 L/min, 17 L/min, and 26 L/min. As mentioned above, 10 L/min represents the maximum flow rate (with oxygen concentration greater than 90%) of mobile medical oxygen generators currently available on the market.

As shown in Fig. 7(d), the incorporation of a scroll compressor into the oxygen generator enhanced its operational efficacy, enabling a consistent output of 17 L/min with an oxygen concentration exceeding 90%. From Table 1, the subjects were able to achieve an increase in oxygen concentration from 37 to 49% at ventilator side. At the flow rate of 26 L/min, the oxygen generator was capable of providing a greater mass flow of oxygen, and the oxygen concentration of the ventilator was further increased to 58%. This indicates that the implementation of a scroll compressor and the designed oxygen supply strategy resulted in a 20% increase in oxygen concentration at the respiratory end. Under the flow rates of 10 L/min and 17 L/min, females obtained significantly higher oxygen concentrations. At the flow rate of 26 L/min, the mean oxygen concentration of the female subjects was also higher, although this did not reach a statistically significant difference, which may be attributed to the relatively small sample size. The higher oxygen concentration observed in females can be attributed to their lower respiratory flow consumption. Furthermore, the ventilator’s performance at a given oxygen concentration was evaluated. At a control target of 50%, the mean oxygen concentration error of each participant was maintained at a level of less than ± 0.2%, with an SD of less than 1.3%.

**Table 1:** Oxygen concentration under different high oxygen concentration air supply and which with a control target of 50%. We did not find any significant difference between sitting and supine in each subject. Statistical significance: *P < .05, **P < .01, N.S, not significant.

Subject	Posture	Output parameters of the oxygen generation module			Oxygen output is 26 L/min, 79%, oxygen concentration is controlled at 50%
		10 L/min, 95%	17 L/min, 93%	26 L/min, 79%	
		Output oxygen concentration (%)			
1 (M)	sitting	37.6 ± 1.5	49.0 ± 1.5	57.9 ± 2.0	49.9 ± 1.2
	supine	37.8 ± 1.6	48.8 ± 1.4	58.1 ± 1.7	50.1 ± 1.1
2 (M)	sitting	36.9 ± 1.4	48.2 ± 1.7	57.4 ± 1.4	49.8 ± 1.3
	supine	37.1 ± 1.4	48.3 ± 1.5	57.7 ± 1.5	50.2 ± 1.2
3 (F)	sitting	38.4 ± 1.7	49.3 ± 2.1	58.3 ± 1.9	49.8 ± 1.3
	supine	38.7 ± 1.9	49.3 ± 1.7	58.3 ± 1.6	49.8 ± 1.2
4 (F)	sitting	38.3 ± 1.8	48.9 ± 1.8	58.2 ± 1.9	50.0 ± 1.2
	supine	38.4 ± 1.9	49.0 ± 1.7	58.1 ± 2.0	50.2 ± 1.3
		F > M**	F > M**	N.S	N.S

### Subject-ventilator respiratory synchronization

**Fig. 8:**
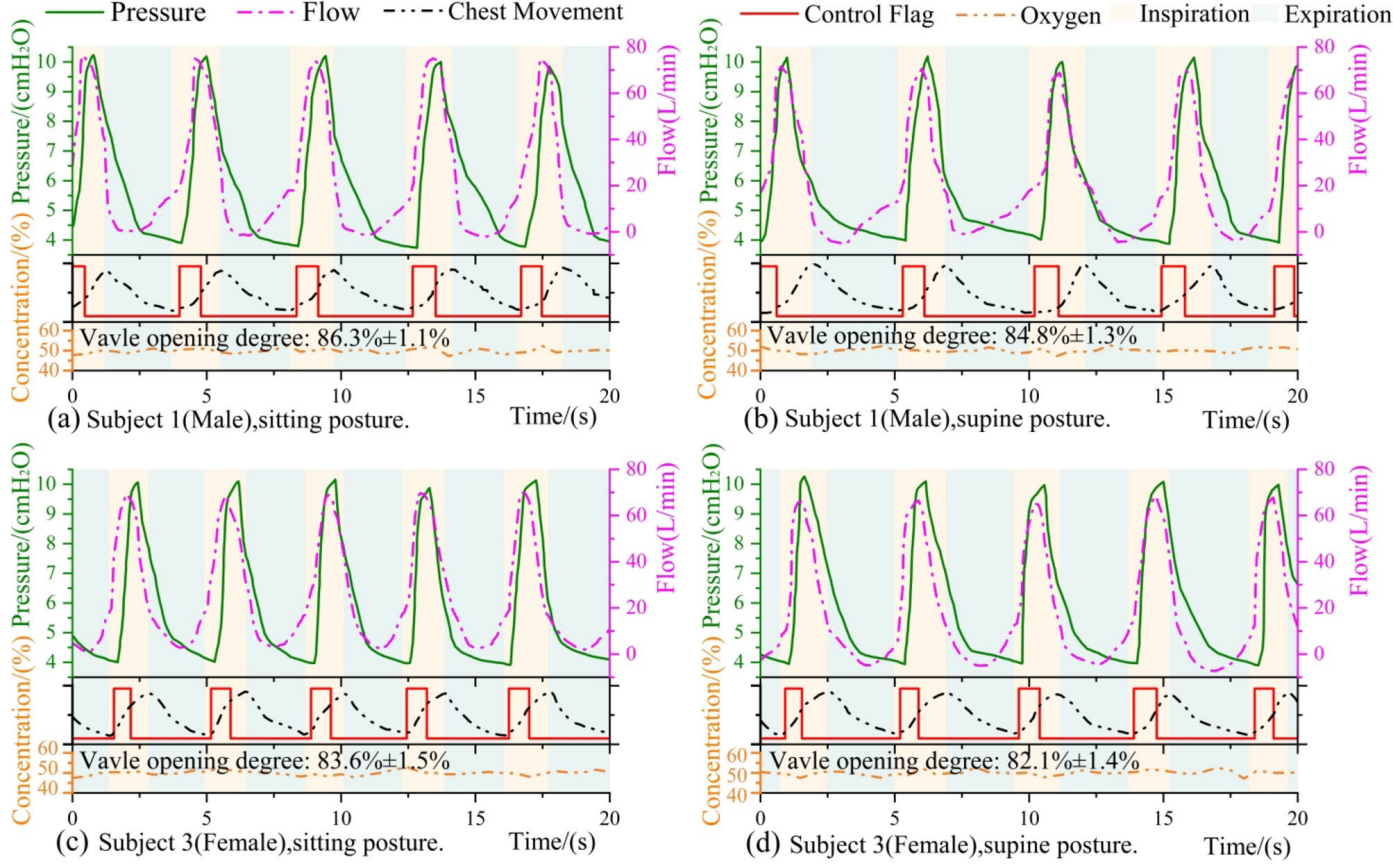
The subject’s respiratory waveform. (**a**) Subject 1(Male) in a sitting posture; (**b**) Subject 1(Male) in a supine posture; (**c**) Subject 3(Female) in a sitting posture; (**d**) Subject 3(Female) in a supine posture. The rotation speed of the compressor is 2000 rpm in the above four ventilation experiments, and the opening degree of the proportional valve (mean and standard deviation calculated over the five minutes of ventilation experiments) are labeled below each subplot.

The respiratory flow rate, pressure, and chest movement (as indicated by the RIP sensor) were recorded simultaneously during the ventilation to estimate respiratory synchronization. The actual times of inspiration and expiration were obtained from the RIP curve. Given the similarity of these curves across subjects, only the partial curves for subject 1 and subject 3 are presented in Fig. 8(a)-(b) and Fig. 8(c)-(d) respectively. The control delays and some respiratory parameters of each subject in the sitting and supine postures were also statistically analyzed. We roughly supposed that the average flow (FS_S_,mean) as the leakage value, and (FS_S_(t) - FS_S_,mean) as the subject’s respiratory flow. The value of (FS_S_(t) - FS_S_,mean) was integrated during inspiratory phases to obtain approximate tidal volume (TV). All ventilation experiments conducted in this section were performed with a target oxygen concentration of 50%.

As can be seen from the curves shown in Fig. 8, the controller successfully identified the onset of the inspiratory phase, and the blower provided timely support air to reach IPAP. As a consequence of the reduction in support pressure resulting from inspiratory effort and the necessity for a response time of the blower, ITDs were larger than ISDs. From Table 2, the difference between the two values was approximately 110 to 130 ms, and the ISD time of the controller was about 62 to 70 ms. No significant difference was observed between sitting and supine postures for each subject, either between males or females in terms of ITD and ISD. The control flag switched after the inspiratory effort and before the actual expiratory phase. Satisfactory respiratory synchronization was achieved, which is manifested as a corresponding pressure supply in both inspiratory and expiratory phases, no expiration pressure overshoot and the supplied air flow was matched with the inspiratory effort. In addition, the oxygen concentration was controlled and essentially stable at around 50%.

**Table 2:** Control delay and respiratory parameters. We did not obtain any significant difference between sitting and supine in each subject in ITD and ISD. These two indices did not show significant differences between males and females either. For TV, RR and I: E, statistical significance between sitting and supine: *P < .05, **P < .01, N.S, not significant.

Subject	Posture	ITD (s)	ISD (s)	TV (ml)		RR (breaths/min)		I: E	
1 (M)	sitting	0.179 ± 0.015	0.069 ± 0.012	724 ± 21	**	14.03 ± 0.38	**	0.635 ± 0.041	*
	supine	0.188 ± 0.018	0.065 ± 0.010	697 ± 17		12.75 ± 0.53		0.664 ± 0.056	
2 (M)	sitting	0.183 ± 0.017	0.067 ± 0.012	716 ± 23	**	14.87 ± 0.39	**	0.625 ± 0.046	N.S
	supine	0.188 ± 0.018	0.066 ± 0.011	689 ± 18		13.91 ± 0.31		0.647 ± 0.053	
3 (F)	sitting	0.190 ± 0.022	0.064 ± 0.011	657 ± 26	**	16.15 ± 0.31	**	0.639 ± 0.055	N.S
	supine	0.195 ± 0.019	0.066 ± 0.012	643 ± 19		13.99 ± 0.19		0.652 ± 0.050	
4 (F)	sitting	0.184 ± 0.016	0.062 ± 0.013	685 ± 24	**	17.21 ± 0.69	**	0.583 ± 0.040	N.S
	supine	0.190 ± 0.018	0.064 ± 0.011	662 ± 21		14.73 ± 0.41		0.605 ± 0.041	
		N.S	N.S	M > F**		F > M**		M > F*	

Additionally, the differences in respiratory rate (RR), inspiration-to-expiration ratio (I: E) and TV between sitting and supine postures and the impact of gender on these variables under BiPAP respiratory support, were also examined. As shown in Table 2, in individuals, the RR is higher when sitting. With regard to I: E in both postures, the three subjects don’t exhibit a statistically significant difference, this may be attributed to the limited sample size. Besides, females exhibit a larger RR, and their I: E is smaller. TV is significantly greater in males than in females, and in the sitting posture compared to the supine posture.

## Discussion

A mobile oxygen generator integrated ventilator was proposed in this study. To the best of our knowledge, this is the first study that combines the design of oxygen generation and respiratory support systems. We employed a scroll compressor for the oxygen generator to significantly increase the oxygen flow of the mobile oxygen generator, and an optimized oxygen supply strategy in combination with the respiratory support system to realize oxygenated respiratory support. The results demonstrate that the integrated ventilator is capable of providing an oxygen concentration above 55% under BiPAP respiratory support without relying on any external oxygen source. The proposed ventilator addresses the dependence on oxygen sources for oxygenated respiratory support, which is of great significance for field rescue, patient transportation, and emergency responses to public events.

The principal sources of medical oxygen include cryogenic distillation, oxygen cylinders, and PSA^5,31^. Cryogenic distillation is an appropriate method for centralized oxygen consumption in large medical institutions. The oxygen needs to be stored in cryogenic tanks and transported via dedicated pipelines^32^. Therefore, its high requirements for construction and maintenance makes it a challenge for mobile cabin hospitals during the COVID-19 pandemic^33,34^. Oxygen cylinders, with the advantages of convenient transportation and many application scenarios, are the most prevalent oxygen source utilized in ambulances and mobile scenes. Whereas, long-term transportation and storage will bring gas loss, and repeated prefusion restricts its continuous application. In addition, high-pressure cylinders also have been identified as a potential risk factor for explosions in military sites^35,36^. By contrast, our integrated ventilator addresses the continuous oxygenated respiratory support in field rescues without the above limitations.

PSA is an appropriate technology for mobile oxygen generation^37,38^. However, the majority of the existing mobile PSA oxygen generators exhibit an oxygen flow rate of 0.5–5 L/min, and up to 10 L/min, making them insufficient to sustain ventilator operation^39^. Although Rubio et al. attempted to address this issue by using two sets of oxygen generators in parallel to achieve a 15 L/min oxygen flow, they did not present any experiment data with the ventilator^18^. In fact, a crucial factor that determines oxygen generation capacity is the type of compressor. For the current oxygen generators that use piston compressors, the internal air leakage and periodic resetting of the piston limit their compression efficiencies. To overcome this limitation, we designed a small scroll compressor for oxygen generators. Without mechanical friction, this approach enhanced compression efficiency and reduces power consumption. Its dynamic gas sealing was realized based on two precision-manufactured, staggered scroll disks. With our redesigned overall structure, our small scroll compressor achieved continuous gas compression flow of 180 L/min, with reduced power consumption and weight (465 W and 6 kg). In comparison, the compressor utilized in the existing 10 L/min oxygen generator has a displacement volume of approximately 120 L/min, with a rated power of 580 W and a weight of about 10 kg.

Using our redesigned scroll compressor combined with the optimized switching time of molecular sieve adsorption and reactivation^15,40^, the proposed scroll compressor-based PSA oxygen generator achieved an oxygen output exceeding 17 L/min with a concentration of 93 ± 3%. Under such oxygen supply parameters, compared to an oxygen supply of 10 L/min, the maximum inspiratory oxygen concentration at the respiratory end of subjects increased from 37 to 48%. Oxygen generators are generally designed to output more than 90% oxygen concentration for direct use by patients. In the market, the air-to-oxygen ratio of most oxygen generators is about 13:1, with an oxygen yield coefficient of 35%^41,42^. The oxygen system we developed can achieve an output of 17 L/min at 93% oxygen concentration with an oxygen yield coefficient of 41.4%. For our integrated ventilator, the oxygen generator is incorporated to provide a greater oxygen content rather than high oxygen concentration. Therefore, we analyzed the relationship between the oxygen generation flow and concentration in different scenarios, and then innovatively used this characteristic flow-concentration curve to match the oxygen supply strategy for the integrated ventilator. Our results showed that the designed oxygen generator can output a flow of 26 L/min with an oxygen concentration of 79% and oxygen yield coefficient increased to 53.7%. Based on such an oxygen supply strategy, the maximum inspiratory oxygen concentration at the respiratory end could be further increased to 58%. The aggregate power consumption of the entire system is less than 700 W. This consumption is considerably lower than the current discharge power of electric vehicles, which typically range from 2 kW to 10 kW. It can achieve oxygenated respiratory support merely through electricity, without external replenishment, and requires little maintenance. Consequently, our integrated ventilator has the potential to reform the current rescue mode in the context of on-site first aid and field rescue.

Synchronization between the subject and the ventilator is a crucial issue in mechanical ventilation. In this study, we triggered in time and ensured a stable transition of the expiratory phase without pressure overshoot to optimize respiratory synchronization. Asynchrony has been demonstrated to have a detrimental impact on patient comfort, and patients may even require sedatives and muscle relaxants to alleviate asynchrony in some cases^43,44^. By monitoring the electrical activity of the patient’s diaphragm, neurally adjusted ventilatory assist (NAVA) enhances subject-ventilator synchronization with a trigger delay time of less than 100 ms^45–47^. However, the placement of the electrodes limits its practical application^48,49^. The delay time of pressure support ventilation based on flow or pressure trigger is typically within the range of 126 ms to 281 ms^49^. The integrated ventilator proposed in this study triggered the blower to provide IPAP by detecting the moment of rapid rise in inspiratory flow, achieving a trigger delay time within 200 ms and a sensing delay time within 70 ms with stable performance. The strategy of early expiratory transition circumvented airflow accumulation in the ventilator line at the outset of expiration without pressure overshoot, thus enhancing the subject’s compliance. As illustrated in the subject’s respiratory curve, when the inspiratory phase was sensed, the pressure did not begin to rise immediately because the subject was still in the inspiratory effort state. However, the rapid rise in flow indicated that the blower had commenced supplying air. Evaluation of the overall waveform of the ventilation process demonstrates that the integrated ventilator effectively tracks flow and pressure in accordance with the subject’s respiratory state^24,50,51^. The system, including the PSA oxygen generation module, the air-oxygen mixing module, and the respiratory support module are closed-loop controlled and adjustable, offering great possibilities for personalized and precise treatment in the future.

The oxygen concentration assessment indicates that the maximum inspiratory oxygen concentration in male subjects was lower than that in females at 10 L/min and 17 L/min oxygen supply, respectively. This may be attributed to the fact that male subjects generally have larger tidal volume than their female counterparts. The respiratory rate during ventilation was different between genders and postures. Moreover, the inspiratory-to-expiratory ratio in the sitting and supine postures had a greater effect on males than on females. These findings are consistent with previous studies which demonstrated significant differences between males and females in respiratory physiology, including lung capacity, respiratory muscle strength, and airway resistance^52^. Males typically have a larger thoracic cavity and greater lung volumes than females. This means that when males are in the supine position, respiratory mechanics and lung compliance are more susceptible to changes in position, and abdominal pressure on the diaphragm may be more pronounced, thus affecting I: E more significantly^53,54^.

Apparently, there are some limitations to our study. Since the proposed integrated ventilator is only a prototype system, it is difficult to implement invasive ventilation under the current conditions. There is a lack of experiments on the effect of ventilation on the respiratory state and tests involving patients with relevant respiratory diseases. Whether the system meets the therapeutic needs of critically ill patients who require a combination of invasive ventilation needs further research. In addition, we will try to design the pressure regulated volume controlled ventilation (PRVCV) mode in the follow-up. The novel component in the designed system is the scroll compressor. We have improved the production process to reduce costs, achieving comparable costs with current swing piston compressors under batch production. At present, the scroll compressor can be used for up to 28,000 h in a laboratory environment, but the reliability in the product needs to be further verified by extensive runs in the real world. Furthermore, we are currently developing larger scroll compressors and oxygen generation systems for higher requirements of oxygen concentration. Meanwhile, achieving more precise oxygen concentration control will depend on the development of novel sensors to some extent. Nevertheless, the preliminary experimental findings from this study may provide valuable insights for further development in this field.

## Conclusion

This study details the design and development of an oxygen generator integrated ventilator intended for use in emergency medical rescue scenarios. The oxygen generation system, comprising an oil-free scroll compressor and molecular sieves, facilitates the generation of mobile, high-flow oxygen. Coupled with an optimized oxygen supply and respiratory synchronization strategy, this system achieves oxygenated BiPAP respiratory support. The oxygen generator integrated ventilator can be employed as a joint control platform for oxygen control and respiratory pressure support to further investigate the impact of oxygen concentration and respiratory support strategies on respiratory characteristics, as well as the treatment of respiratory diseases.

## Data Availability

The datasets used and/or analyzed during the current study are available from the corresponding author on reasonable request.

## References

[1] Dolinay, T., Jun, D., Chen, L. & Gornbein, J. Jun, Mechanical ventilator liberation of patients with COVID-19 in Long-term acute care hospital, chest., **161**, 6, pp. 1517–1525, (2022). 10.1016/j.chest.2022.02.030 PMC8875856 35227663

[2] Rochwerg, B. et al. Official ERS/ATS clinical practice guidelines: noninvasive ventilation for acute respiratory failure. *Eur. Respir. J.* **50** (2), 1 (2017). Art. 1602426. 10.1183/13993003.02426-2016 28860265

[3] Lockey, D. J. et al. Mar., AAGBI: Safer pre-hospital anaesthesia 2017: Association of Anaesthetists of Great Britain and Ireland, Anaesthesia., vol. 72, no. 3, pp. 379–390, (2017). 10.1111/anae.13779 PMC5324693 28045209

[4] Epstein, S. K. Weaning from ventilatory support. *Curr. Opin. Crit. Care*. **15** (1), 36–43 (Feb 2009). 10.1097/MCC.0b013e3283220e07 19179869

[5] Ismail, J. & Bansal, A. Medical oxygen: A lifesaving drug during the COVID-19 Pandemic-Source and distribution. *Indian J. Pediatr.* **89** (6), 607–615 (Jun 2022). 10.1007/s12098-021-03978-0 PMC8758929 35029808

[6] Darwood, A., McCanny, J., Kwasnicki, R., Martin, B. & Jones, P. Nov, The design and evaluation of a novel low-cost portable ventilator, anaesthesia., **74**, 11, pp. 1406–1415, (2019). 10.1111/anae.14726 31161650

[7] Truog, R. D., Mitchell, C. & Daley, G. Q. The toughest Triage - Allocating ventilators in a pandemic. *N. Engl. J. Med.*, **382**, 21, pp. 1973–1975, May 21 2020. 10.1056/NEJMp2005689 32202721

[8] Zhang, H. L. et al. Strategy of medical rescue during Wenchuan earthquake. *J. Clin. Anesthesiology*. **11** (1), 53–55 (2009).

[9] Powell, T., Christ, K. C. & Birkhead, G. S. Allocation of ventilators in a public health disaster. *Disaster Med. Pub. Health Prep.* **2** (1), 20–26 (2008). 18388654 10.1097/DMP.0b013e3181620794

[10] Ma, Y. et al. Experience on rescue of patients with severe multiple injuries after sudden disaster under field condition. *Acad. J. Second Military Med. Univ.* **29** (6), 583–585 (2008).

[11] Yun, T. A. O., He, H. & Ping, F. Anesthetic management of field treatment for traumatic patients in Wenchuan earthquake. *J. Clin. Anesthestology*. **25** (3), 225–227 (2009).

[12] Blakeman, T., Fowler, J. M., Salvator, A. & Rodriquez, D. Maximizing Oxygen Delivery in Portable Ventilators, Military Medicine., vol. 188, no. 7–8, pp. E1717-E1722, Jul-Aug 2023. 10.1093/milmed/usab561 35134973

[13] Bordes, J., Savoie, P. H., Ferreres, J., Celindano, F. & Kaiser, E. FiO2 delivered by a turbine portable ventilator with an oxygen concentrator in an austere environment. *Med. Et Armees*. **42** (5), 447–450 (Dec 2014). 10.1016/j.jemermed.2014.04.033 24950943

[14] Shrivastava, S., Verma, A., Ramkumar, J. & Aryal, R. A comprehensive study for improving the working parameters for the design of a PSA-based oxygen concentrator, Engineering Research Express., vol. 6, no. 1, Mar 1 Art. no. 015025. (2024).

[15] Zhang, Q. et al. Experimental study on oxygen concentrator with wide product flow rate range: individual parametric effect and process improvement strategy. *Sep. Purif. Technol.*, **274**, Nov 1 2021, Art. 118918.

[16] Yadav, V. K. et al. Recent trends in the nanozeolites-based oxygen concentrators and their application in respiratory disorders. *Front. Med.*, **10**, Apr 27 2023, Art. 1147373. 10.3389/fmed.2023.1147373 PMC10174459 37181347

[17] Nair, T. S., Kumar, S. V., Harikumar, M. E. & Santhosh kumar, C. An experimental study on modification of oxygen concentrator components for better purity, in 2022 International Conference on Distributed Computing, VLSI, Electrical Circuits and Robotics, Shivamogga, India, pp. 223–228. (2022).

[18] Rubio, J. et al. Mar., COVOX: providing oxygen during the COVID-19 health emergency, hardwarex., **13**, (2023). Art. no. e00383. 10.1016/j.ohx.2022.e00383 PMC9763216 36568708

[19] Sun, J., Peng, B., Zhu, B. & Li, Y. Analysis of tangential leakage flow characteristics of Oil-Free scroll expander for a Micro-Scale compressed air energy storage system, entropy., **25**, 2, Feb 2023, Art. 339. 10.3390/e25020339 PMC9955695 36832705

[20] Lu, R. J. et al. Discussion and practice on safety improvement of medical compressed air system. *China Med. Devices*. **36** (3), 58–61 (2021).

[21] Blanch, L. et al. Asynchronies during mechanical ventilation are associated with mortality. *Intensive Care Med.* **41** (4), 633–641 (Apr 2015). 10.1007/s00134-015-3692-6 25693449

[22] Carteaux, G. et al. Comparison between neurally adjusted ventilatory assist and pressure support ventilation levels in terms of respiratory effort. *Crit. Care Med.*, **44**, 3, pp. 503–511, Mar 2016. 10.1097/CCM.0000000000001418 26540399

[23] Doorduin, J. et al. Sep, Respiratory muscle effort during expiration in successful and failed weaning from mechanical ventilation, anesthesiology., **129**, 3, pp. 490–501, (2018). 10.1097/ALN.0000000000002256 29771711

[24] Hao, L. et al. A novel method to evaluate Patient-Ventilator synchrony during mechanical ventilation. *Complexity* **2020** (Sep 15), Art4828420 (2020).

[25] Nakornnoi, B., Tscheikuna, J. & Rittayamai, N. May, The effects of real-time waveform analysis software on patient ventilator synchronization during pressure support ventilation: a randomized crossover physiological study. *BMC Pulm. Med.*, **24**, 1, (2024). 10.1186/s12890-024-03039-0 PMC11064376 38693506

[26] Chen, Y., Cheng, K. & Zhou, X. Performance characteristics of seven bilevel mechanical ventilators in Pressure-Support mode with different cycling criteria: A comparative bench study. *Med. Sci. Monit.* **21**, 310–317 (Jan 2015). 10.12659/MSM.892080 PMC4315647 25619202

[27] Carteaux, G. et al. Aug., Patient-Ventilator Asynchrony During Noninvasive Ventilation, Chest, vol. 142, no. 2, pp. 367–376, (2012). 10.1378/chest.11-2279 22406958

[28] Jiang, D. & Huang, F. Influence Factors of Pressure Swing Adsorption for Oxygen Production by the Orthogonal Method and the Response Surface Method, Processes, vol. 12, no. 7, p. 1306, Jun. (2024).

[29] Li, Y. et al. Sep., Experimental Study on Vehicle Pressure Swing Adsorption Oxygen Production Process Based on Response Surface Methodology, Separations, vol. 11, no. 9, pp. 267–267, (2024).

[30] Jiang, D. & Li, H. Study on Influencing Factors of Molecular Sieve Oxygen-Production System, Processes, vol. 11, no. 1, p. 124, Jan. (2023).

[31] Jain, R. & Sharma, C. Oxygen Supply in Hospitals: Requisites in the Current Pandemic, Anesthesia, essays and researches., vol. 15, no. 3, pp. 253–256, (2021). 10.4103/aer.aer_116_21 PMC8936862 35320955

[32] Allam, R. J. Improved oxygen production technologies, in 9th International Conference on Greenhouse Gas Control Technologies, Washington, DC, pp. 461–470. (2009).

[33] Ritz, R. H. & Previtera, J. E. Oxygen supplies during a mass casualty situation, respiratory care., **53**, 2, pp. 215–224, Feb 2008. 18218152

[34] Blakeman, T. C. & Branson, R. D. *Oxygen Supplies Disaster Manage. Respiratory Care*, **58**, 1, 173–182, Jan (2013). 23271827 10.4187/respcare.02088

[35] Rybak, M. et al. Ultraportable oxygen concentrator use in US army special operations forward area surgery: A proof of concept in multiple environments, military medicine., **182**, no. 1–2, pp. E1649-E1652, Jan-Feb 2017. 10.7205/MILMED-D-16-00100 28051988

[36] Nowadly, C. D., Portillo, D. J., Davis, M. L., Hood, R. L. & De Lorenzo, R. A. The Use of Portable Oxygen Concentrators in Low-Resource Settings: A Systematic Review, Prehospital and Disaster Medicine., vol. 37, no. 2, pp. 247–254, Apr (2022). 10.1017/S1049023X22000310 35232523

[37] Oxygen sources and distribution for COVID-19 treatment centers, World Health Organization. (2020). Available at: https://www.who.int/publications/i/item/oxygen-sources-and-distribution-for-covid-19-treatment-centres (Accessed: 5 February 2024).

[38] Qadir, S. et al. Experimental and Numerical Analysis on the Enhanced Separation Performance of a Medical Oxygen Concentrator through Two-Bed Rapid Pressure Swing Adsorption, Industrial & Engineering Chemistry Research., vol. 60, no. 16, pp. 5903–5913, Apr 28 2021.

[39] Hardavella, G., Karampinis, I., Frille, A., Sreter, K. & Rousalova, I. Oxygen devices and delivery systems, breathe., **15**, 3, p. E108 -E116, Sep 1 (2019). 10.1183/20734735.0204-2019 PMC6876135 31777573

[40] Zhu, X., Liu, Y., Yang, X., Liu, W. & Li, Y. Effect of adsorption and desorption pressure on the velocity and cycling performance of rapid pressure swing adsorption. *Chin. J. Eng.* **38** (7), 993–1001 (2016).

[41] Vemula, R. R., Urich, M. D. & Kothare, M. V. Experimental design of a Snap-on and standalone single-bed oxygen concentrator for medical applications. *Adsorption-Journal Int. Adsorpt. Soc.* **27** (4), 619–628 (May 2021). 10.1007/s10450-021-00299-8 PMC7882046 33612972

[42] Zhu, X., Liu, Y. & Yang, R. T. Effects of operating temperature on the performance of small scale rapid cycle pressure swing adsorption air separation process. *Adsorption-Journal Int. Adsorpt. Soc.* **27** (2), 205–212 (Feb 2021).

[43] Subira, C., de Haro, C., Magrans, R., Fernandez, R. & Blanch, L. Minimizing Asynchronies in Mechanical Ventilation: Current and Future Trends, Respiratory Care., vol. 63, no. 4, pp. 464–478, Apr 1 (2018). 10.4187/respcare.05949 29487094

[44] Liu, K., Chen, Y., Lin, R. & Han, K. Clinical features of COVID-19 in elderly patients: A comparison with young and middle-aged patients. *J. Infect.*, **80**, 6, pp. E14-E18, Jun 2020. 10.1016/j.jinf.2020.03.005 PMC7102640 32171866

[45] Yonis, H. et al. Patient-ventilator synchrony in neurally adjusted ventilatory assist (NAVA) and pressure support ventilation (PSV): a prospective observational study. *BMC Anesthesiol.*, **15**, Aug 8 2015, Art. 117. 10.1186/s12871-015-0091-z PMC4528778 26253784

[46] Terzi, N. et al. Clinical review: Update on neurally adjusted ventilatory assist-report of a round-table conference, Crit Care., vol. 16, no. 3, p. 225, Jun 20., (2012). 10.1186/cc11297 PMC3580602 22715815

[47] Beloncle, F. et al. A diaphragmatic electrical activity-based optimization strategy during pressure support ventilation improves synchronization but does not impact work of breathing. *Crit. Care*. **21** (1), 21 (Jan 31 2017). 10.1186/s13054-017-1599-z PMC5282691 28137269

[48] Patroniti, N. et al. Respiratory pattern during neurally adjusted ventilatory assist in acute respiratory failure patients. *Intensive Care Med.* **38** (2), 230–239 (Feb 2012). 10.1007/s00134-011-2433-8 22127483

[49] Liu, L. et al. Neural control of pressure support ventilation improved patient-ventilator synchrony in patients with different respiratory system mechanical properties: a prospective, crossover trial. *Chin. Med. J.*, **134**, 3, pp. 281–291, Feb 5 2021. 10.1097/CM9.0000000000001357 PMC7846453 33470654

[50] Mojoli, F., Iotti, G., Arnal, J. M. & Braschi, A. Is the ventilator switching from inspiration to expiration at the right time? Look at waveforms! *Intensive Care Med.*, **42**, 5, pp. 914–915, May 2016. 10.1007/s00134-015-4174-6 26690075

[51] Doerschug, K. C. Patient-Ventilator synchrony. *Clin. Chest. Med.* **43** (3), pp511–518 (Sep 2022). 10.1016/j.ccm.2022.05.005 36116818

[52] LoMauro, A. & Aliverti, A. Sex differences in respiratory function. *Breathe* **14** (2), 131–140 (May 2018). 10.1183/20734735.000318 PMC5980468 29875832

[53] Ides, K. M. et al. Dec., The effect of posture on airflow distribution, airway geometry and air velocity in healthy subjects, BMC Pulmonary Medicine, vol. 22, no. 1, (2022). 10.1186/s12890-022-02276-5 PMC9753395 36522658

[54] Shaghayeghfard, B., Karimi, M. T., Abbasi, L. & Razeghi, M. Respiratory Function Assessment through Kinematic Analysis of Chest Wall Movements: Effects of Position and Gender, Journal of biomedical physics and engineering, vol. online, Jan. (2023). 10.31661/jbpe.v0i0.2105-1335 PMC10862111 38357601

